# 

**Published:** 1893-05

**Authors:** 


### CONTENTS—MAY, 1893.—VOL. VIII., NO. 11.

ORIGINAL COMMUNICATIONS—

Radical Cure for Inguinal Hernia. Prof. James E. Thompson, Galveston . . . 431

A Plea for Conservatism in Gynecology. D. R. Wallace, M. D., L.L.D., Waco . . . 441

ABSTRACTS OF CURRENT MEDICAL LITERATURE—

Department of Therapeutics. Dr. David Cerna, Galveston . . . 447

Department of Practice of Medicine. Prof. A. J. Smith, Galveston . 450

St. Louis Clinic. Dr. Z. F. Lillard, Tyler . . . 453

SOCIETY NOTES—

Texas State Medical Association . . . 455

EDITORIAL—

Paragraphs of Interest . . . 480

MEDICAL NEWS AND MISCELLANY—

Many Interesting Items . . . 482

BOOK NOTICES—

The International Medical Annual . . . 485

PUBLISHERS' NOTES . . . 485

